# Chromium Propionate Enhances Adipogenic Differentiation of Bovine Intramuscular Adipocytes

**DOI:** 10.3389/fvets.2015.00026

**Published:** 2015-09-08

**Authors:** Rebecca J. Tokach, Flavio R. B. Ribeiro, Ki Yong Chung, Whitney Rounds, Bradley J. Johnson

**Affiliations:** ^1^Department of Animal and Food Sciences, Texas Tech University, Lubbock, TX, USA; ^2^Hanwoo Research Institute, National Institute of Animal Science, Rural Development Administration, Kangwon, South Korea; ^3^Kemin Animal Nutrition and Health North America, Des Moines, IA, USA

**Keywords:** beef cattle, chromium, GLUT4, marbling

## Abstract

*In vitro* experiments were performed to determine the effects of increasing concentrations of chromium propionate (CrPro) on mRNA and protein abundance of different enzymes and receptors. Intramuscular (IM) and subcutaneous (SC) preadipocytes and bovine satellite cells were isolated from the longissimus muscle to determine the effect of treatment on glucose transporter type 4 (GLUT4) and peroxisome proliferator-activated receptor γ mRNA and GLUT4 protein abundance. Preadipocyte cultures were treated with differentiation media plus either sodium propionate or different concentrations of CrPro for 96, 120, and 144 h before harvest. This study indicated that adipogenesis of the bovine IM adipocytes were more sensitive to the treatment of CrPro as compared to SC adipocytes. Enhancement of adenosine monophosphate-activated protein kinase and GLUT4 mRNA by CrPro treatment may enhance glucose uptake in IM adipocytes. CrPro decreased GLUT4 protein levels in muscle cell cultures suggesting that those cells have increased efficiency of glucose uptake due to exposure to increased levels of CrPro. In contrast, each of the two adipogenic lines had opposing responses to the CrPro. It appeared that CrPro had the most stimulative effect of GLUT4 response in the IM adipocytes as compared to SC adipocytes. These findings indicated opportunities to potentially augment marbling in beef cattle fed CrPro during the finishing phase.

## Introduction

It has been generally accepted that chromium (Cr) potentiates the effects of insulin (1). Chromium, included in the diet as KemTRACE^®^_brand_ Chromium Propionate 0.04% (KT Cr), has improved the performance of beef cattle. These improvements have been expressed as additional body weight gain, better feed efficiency, lower morbidity, and mortality (2–4). Initial results from studies with feeder cattle suggest that feeding KT Cr also has positive effects on immune response, cytokines, and acute phase response of cattle to stress (5). The potential effects of improved glucose metabolism on carcass characteristics have shown mixed results (2, 4). The effect of chromium propionate (CrPro) on bovine muscle, intramuscular (IM) adipose, and subcutaneous (SC) adipose tissue development is unknown. This research was conducted to further investigate the effects of CrPro on enhancing adipogenic differentiation of bovine muscle derived cells, IM, and SC adipocytes.

## Materials and Methods

### Preadipocyte and satellite cell isolation from tissues

**Figure 1:**
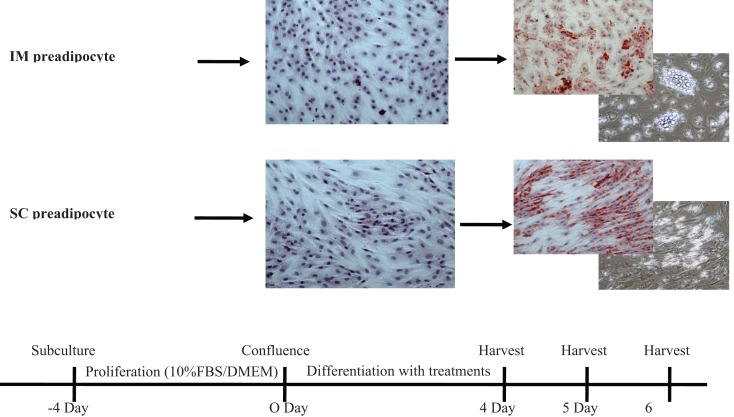
**Time frames of bovine intramuscular (IM) and subcutaneous (SC) preadipocyte differentiation in primary cell culture**. These intramuscular and subcutaneous adipocytes showed different patterns of accumulated lipid droplets. FBS, fetal bovine serum; DMEM, Dulbecco’s modified eagle medium.

Bovine muscle satellite cells (BSC) and preadipocytes were isolated from four 16-month-old British × Continental crossbred steers. Cattle were harvested by captive bolt followed by exsanguination. All experimental procedures were approved by the Texas Tech University Institutional Animal Care and Use Committee, and performed at the Texas Tech University Beef Cattle Research Center. Immediately after hide removal, a section of the longissimus muscle was removed from between the fifth and eighth thoracic ribs and transported to the cell culture laboratory. The following procedures were conducted in a sterile field under a tissue culture hood. For BSC isolation, connective and adipose tissues were removed from the muscle sample (Figure 1). The muscle sample was passed through a sterile meat grinder. The ground muscle was incubated with 0.1% pronase (Calbiochem, La Jolla, CA, USA) in Earl’s Balanced Salt Solution (Sigma, St. Louis, MO, USA) for 1 h at 37°C with mixing every 10 min. The mixture was centrifuged at 1,500 × *g* for 4 min at room temperature following incubation. The pellet that was formed during centrifugation was suspended in phosphate buffered saline (PBS; Invitrogen, Grand Island, NY, USA; 140 mM NaCl, 1 mM KH_2_PO_4_, 3 mM KCl, 8 mM Na_2_HPO_4_), and the suspension was centrifuged at 500 × *g* at 20°C for 10 min. The supernatant was collected and centrifuged at 1,500 × *g* for 10 min at 20°C to pellet the mononucleated cells. Two additional PBS washes and differential centrifugations were conducted before the resulting mononucleated cell preparation was suspended in cold (4°C) Dulbecco’s Modified Eagle Medium (DMEM; Invitrogen) containing 10% fetal bovine serum (FBS; Invitrogen) and 10% (vol/vol) dimethylsulfoxide (Sigma, St. Louis, MO). Cells were stored frozen in liquid nitrogen for future use.

The IM and SC adipose tissues were separated from the muscle, finely minced, and placed in separate containers containing isolation buffer, which consisted of DMEM, collagenase (Sigma, St. Louis, MO, USA), and bovine serum albumin (BSA; Sigma St. Louis, MO, USA). The containers were then incubated in a shaking incubator for 40 min at 38°C. Following incubation, the isolation buffers containing the IM or SC adipose tissue samples were passed through sterilized nylon mesh. Samples were then centrifuged for 5 min at 1,500 rpm. Supernatant was removed and the cell pellet was suspended in 20 mL of warm (37°C) DMEM containing 10% FBS. The centrifugation step was repeated two additional times before the resulting cell pellet was suspended in cold (4°C) DMEM containing 10% FBS and 10% (vol/vol) dimethylsulfoxide. Cells were stored frozen in liquid nitrogen for future use.

### Differentiation of BSC and preadipocyte cultures

Bovine satellite cells and the IM and SC preadipocyte cultures were plated in DMEM containing 10% FBS. Bovine satellite cell cultures were rinsed with DMEM with 10% FBS at 24 and 72 h of incubation. At 120 h of incubation, the BSCs were treated with differentiation media containing 3% horse serum (Sigma, St. Louis, MO, USA), 1.5 μg/mL of BSA-linoleic acid, and one of five treatments. The treatments for the BSC were as follows: (1) control, (2) 0.1 μM CrPro (Kemin Animal Nutrition and Health North America, Des Moines, IA, USA), (3) 1 μM CrPro, (4) 10 μM CrPro, (5) 10 μM sodium propionate (NaPro; Sigma, St. Louis, MO, USA). Chromium for this study was prepared from KemTRACE^®^_brand_ CrPro base (lot # 1006101421), assayed to contain 8.59% Cr. A 100 μM solution was prepared from the above base and was utilized in this *in vitro* study.

Intramuscular and SC preadipocyte cultures were incubated until cells reached approximately 100% confluence. When 100% confluence was achieved, cultures were rinsed three times with serum-free DMEM and DMEM containing 5% FBS plus treatments were added for 96, 120, or 144 h. The treatments for IM and SC preadipocyte cultures were as follows: (1) control, (2) 1 μM CrPro, (3) differentiation media, (4) differentiation media + 0.1 μM CrPro, (5) differentiation media + 1 μM CrPro, (6) differentiation media + 10 μM CrPro, and (7) differentiation media + 10 μM NaPro. The differentiation media used in treatments 3–7 consisted of 10 μM ciglitizone (Sigma, St. Louis, MO, USA), 100 μM oleic acid (Sigma, St. Louis, MO, USA), 1 μM dexamethasone (Sigma, St. Louis, MO, USA), and 10 μM insulin (Sigma, St. Louis, MO, USA). Oil-Red-O and hematoxylin staining were used to confirm the accumulation of lipid droplets in differentiated BSC and IM and SC preadipocyte cultures. The cells were fixed with 10% neutral buffer formalin. After fixation, the cultures were stained with 0.5% Oil-Red-O solution in darkness. Following Oil-Red-O staining, cultures were stained with Harris’ hematoxylin for 30 min in darkness. Cell cultures were mounted in 60% propylene glycol and stored for analysis. IM and SC adipocytes were identified by the presence of lipid droplets in the cytosol, which were changed to a red color by Oil-red-O staining, and hematoxylin staining, which stained the nuclei of cells, was used to identify the multinucleated myotubes of differentiated muscle cells.

### Real-time quantitative-PCR

RNA from cultured BSC and preadipocytes (IM and SC) were isolated with 500 μL of ice-cold buffer containing TRI reagent (Sigma, St. Louis, MO, USA). Concentration of RNA was determined by absorbance at 260 nm. Real-time quantitative-PCR (RTQ-PCR) was used to measure the quantity of glucose transporter type 4 (GLUT4), CCAAT/enhancer-binding protein β (C/EBPβ), peroxisome proliferator-activated receptor γ (PPARγ), stearoyl-CoA desaturase (SCD), and G protein coupled receptor 43 (GPR43) mRNA relative to the quantity of ribosomal protein subunit 9 (RPS9) mRNA in total RNA isolated from BSC and preadipocyte cultures. The quantity of adenosine monophosphate-activated protein kinase (AMPKα), GLUT4, myosin heavy chain (MHC)-I, MHC-IIa, and MHC-IIX, β1-adernergic receptor (AR), and β2-AR relative mRNA was measured relative to the quantity of RPS9 in total RNA isolated from the bovine satellite cells. Assays were performed in the GeneAmp 7900H Sequence Detection System (Applied Biosystems) using thermal cycling parameters recommended by the manufacturer (40 cycles of 15 s at 95°C and 1 min at 60°C). The endogenous RPS9 mRNA control was used to normalize the expression of GLUT4, PPARγ, C/EBPβ, SCD, and GPR43.

### Western blotting

Protein from cultured BSC and preadipocytes (IM and SC) was isolated with 200 μL of ice-cold buffer containing M-PER (Fisher Scientific, Fair Lawn, NJ, USA), protein inhibitor (Roche, Indianapolis, IN, USA), and 2 mM Na_3_VO_4_ (Fisher Scientific). Cell homogenates were mixed with an equal volume of 2× standard SDS sample loading buffer (Invitrogen) and loaded onto gels. Gradient gels (10–20%) were used for SDS-PAGE separation of proteins. Membranes were incubated overnight at 4°C with primary antibodies (Abcam, Cambridge, MA, USA) at a dilution of 1:1,000 in TBS-Tween. Membranes were then incubated with horseradish peroxidase-conjugated secondary antibodies at 1:2,000 dilution for 2 h. After three 10-min washes, membranes were visualized using enhanced chemiluminescent substrate (ECL) Western blotting reagents (Amersham Bioscience, Piscataway, NJ, USA) and exposure to film (MR, Kodak, Rochester, NY, USA). Density of bands was quantified using Imager Scanner II and ImageQuant TL software. To reduce the variation between blots, tissue lysates of both groups were run in a single gel. Band density was normalized according to the glyceraldehyde 3-phosphate dehydrogenase (GAPDH) content.

### Statistical analysis

Data were analyzed as a complete randomized block design using the Mixed procedure (PROC MIXED) of SAS (SAS Institute, Cary, NC, USA). Differences between control and treatment were determined using the least significant difference procedure. Means were considered significantly different at *P* < 0.05.

## Results

**Figure 2:**
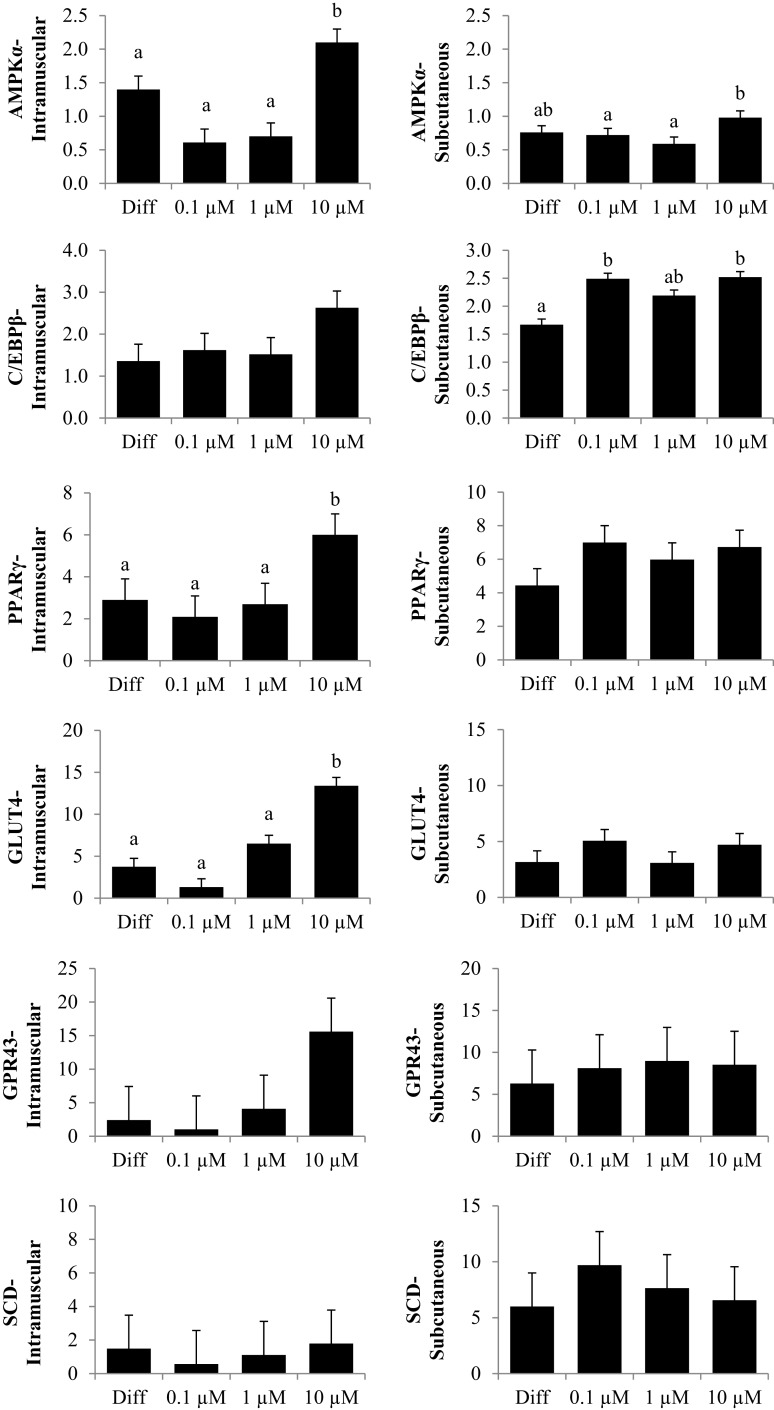
**The effect of dose-dependent treatment of chromium propionate (CrPro) on mRNA abundance of key genes regulating differentiation in intramuscular (IM; left) and subcutaneous (SC; right) adipocytes at 96 h post treatment**. Chromium propionate regulated mRNA levels of adenosine monophosphate-activated protein kinase (AMPKα), CCAAT/enhancer-binding protein β (C/EBPβ), glucose transporter type 4 (GLUT4), G protein coupled receptor 43 (GPR43), peroxisome proliferator-activated receptor γ (PPARγ), and stearoyl-CoA desaturase (SCD) in the IM and SC adipocytes cultures in a dose-dependent fashion. Assays per treatment, *n* = 3. Treatments with different superscripts differ at *P* < 0.05.

The first experiment used RTQ-PCR analysis of IM and SC adipocytes to examine the effect of a 96 h-treatment of CrPro on preadipocyte differentiation. RTQ-PCR analysis of IM cells revealed that 10 μM CrPro increased AMPKα, PPARγ mRNA, and GLUT 4 abundance compared to all other treatments (Figure 2).

These data indicated that the highest dose of CrPro (10 μM) enhanced IM preadipocyte differentiation as evidenced by the increased PPARγ mRNA levels whereas the SC preadipocytes were non-responsive to CrPro in regards to PPARγ concentrations. SC adipocytes treated with 10 μM CrPro had an increased AMPKα mRNA abundance compared to 0.1 and 1 μM CrPro treatments, but did not differ from the differentiated control cultures (Figure 2). To verify that these differences between IM and SC adipocytes were a result of Cr and not its carrier, propionate (Pro), a second experiment was conducted with additional treatments.

**Figure 3:**
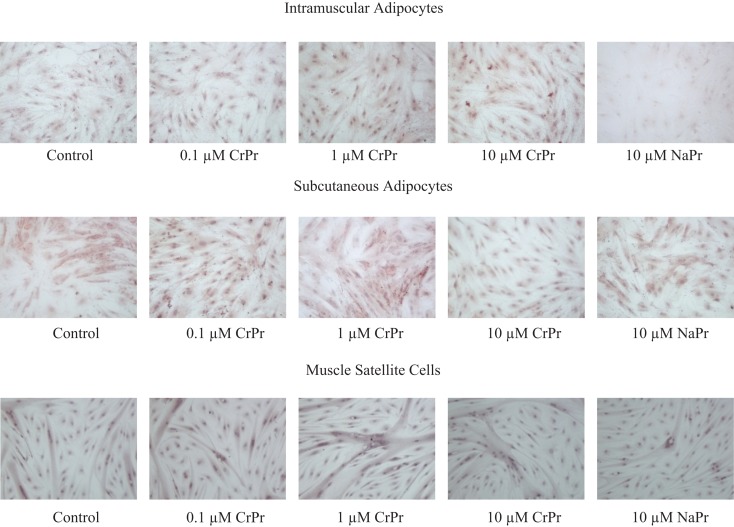
**Morphological analysis of dose-dependent treatment of chromium propionate (CrPro) in intramuscular (IM), subcutaneous (SC) adipocyte, and satellite cell cultures at 96 h post treatment**. Lipid droplets were stained with Oil-Red-O and nuclei were stained with hematoxylin. Increasing Cr dose on IM adipocytes increases lipid droplet numbers. Lipid droplets increase in SC adipocytes up to 1 μM CrPr. Increasing Cr dose in muscle satellite cells results in increasing muscle fiber size and number.

**Figure 4:**
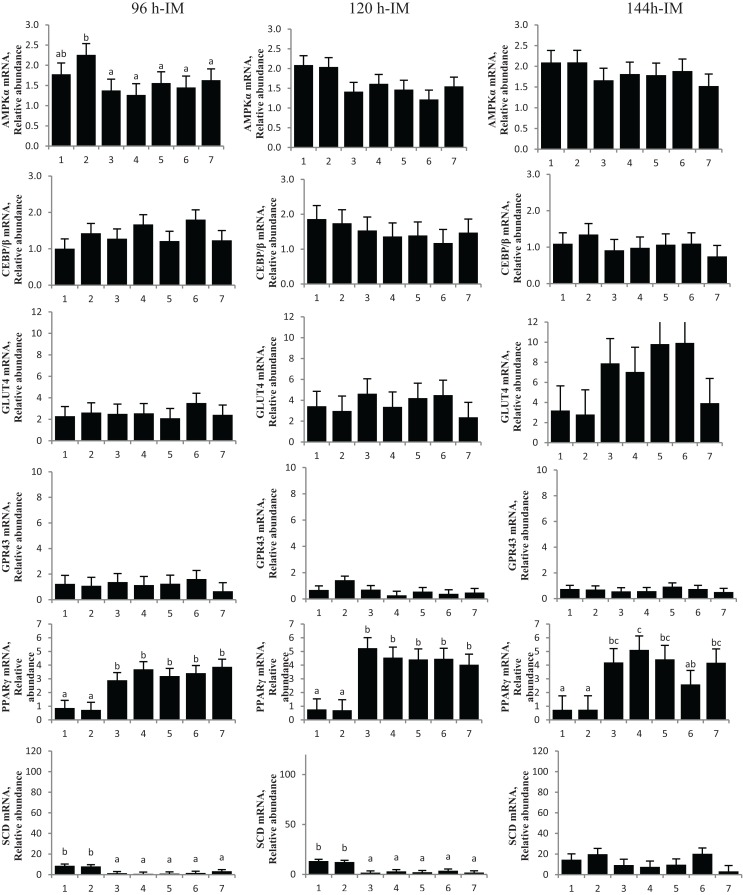
**Chromium propionate (CrPro) regulated mRNA levels of adenosine monophosphate-activated protein kinase (AMPK***α***), CCAAT/enhancer-binding protein ***β*** (C/EBP***β***), glucose transporter type 4 (GLUT4), G protein coupled receptor 43 (GPR43), peroxisome proliferator-activated receptor ***γ*** (PPAR***γ***), and stearoyl-CoA desaturase (SCD) in the intramuscular (IM) adipocytes cultures**. Treatments: (1) 5% fetal bovine serum/Dulbecco’s Modified Eagle Medium (FBS/DMEM; Cont); (2) Cont + 1 μM CrPro; (3) differentiation media (Diff; 5% FBS/DMEM, 10 μg/mL insulin,1.0 μg/mL dexamethasone, 10 μM ciglitizone, 100 μM oleic acid; Diff); (4) Diff + 0.1 μM CrPro; (5) Diff + 1 μM CrPro; (6) Diff + 10 μMCrPro; (7) Diff + 10 μM NaPro. ^abc^Within time period, treatments with different superscripts differ (*P* < 0.05).

In the second experiment, an addition of a 10 μM NaPro treatment helped to verify that the effects previously seen were due to the addition of elemental Cr rather than an effect of Pro. Furthermore, IM and SC adipocytes were differentiated with their respective treatments for 96, 120, or 144 h to observe potential time course effects throughout differentiation. Morphological analysis of these cells revealed that primary cultures of preadipocytes treated with the adipogenic differentiation cocktail accumulated lipid droplets in the cell cytosol after 96 h (Figure 1). Lipid droplets in the IM and SC preadipocyte cultures increased in a dose-dependent manner when treated with CrPro compared to the control and NaPro treatments (Figure 3). However, our qualitative data suggest that a higher dose of Cr may be required to activate lipid filling of IM adipocytes as compared to SC adipocytes as indicated in Figure 4. Morphological analysis for BSC (muscle cells) revealed that treatment of CrPro appeared to increase abundance and diameter of myotubes compared to the control and NaPro-treated cultures (Figure 3). These data would indicate a positive effect on skeletal muscle hypertrophy (size of myotubes) and differentiation (extent and number of myotubes) caused by CrPro addition to these primary cultures of muscle satellite cells.

**Figure 5:**
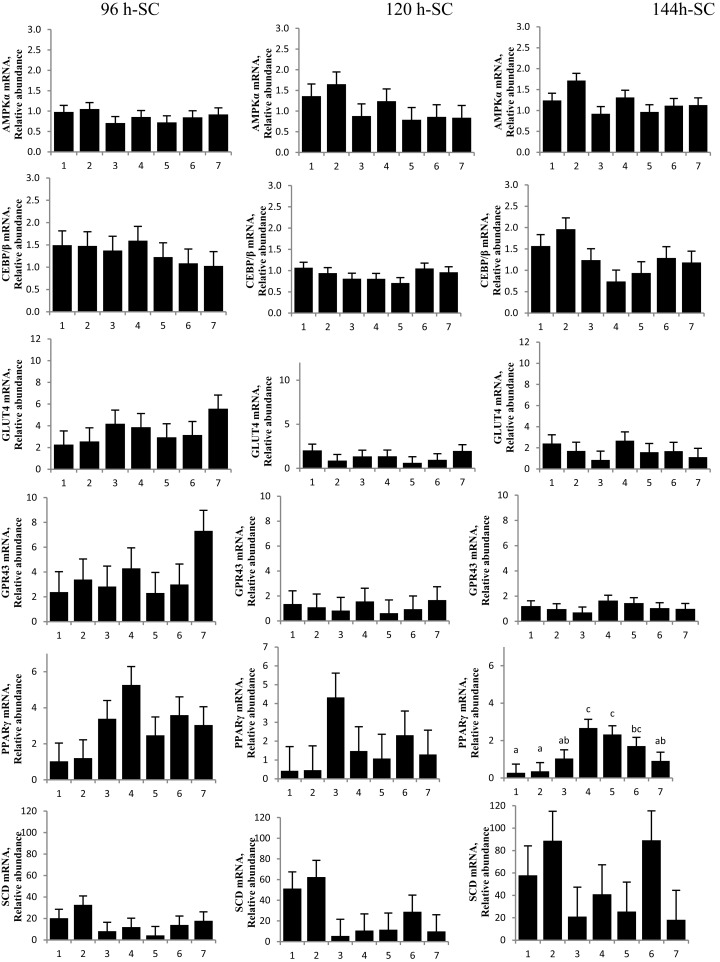
**Chromium propionate (CrPro) regulated mRNA levels of adenosine monophosphate-activated protein kinase (AMPK***α***), CCAAT/enhancer-binding protein ***β*** (C/EBP***β***), glucose transporter type 4 (GLUT4), G protein coupled receptor 43 (GPR43), peroxisome proliferator-activated receptor ***γ*** (PPAR***γ***), and stearoyl-CoA desaturase (SCD) in the subcutaneous (SC) adipocytes cultures**. Treatments: (1) 5% fetal bovine serum/Dulbecco’s Modified Eagle Medium (FBS/DMEM; Cont); (2) Cont + 1 μM CrPro; (3) differentiation media (Diff; 5% FBS/DMEM, 10 μg/mL insulin, 1.0 μg/mL dexamethasone, 10 μM ciglitizone, 100 μM oleic acid; Diff); (4) Diff + 0.1 μM CrPro; (5) Diff + 1 μM CrPro; (6) Diff + 10 μMCrPro; (7) Diff + 10 μM NaPro.^abc^ Within time period, treatments with different superscripts differ (*P* < 0.05).

**Figure 6:**
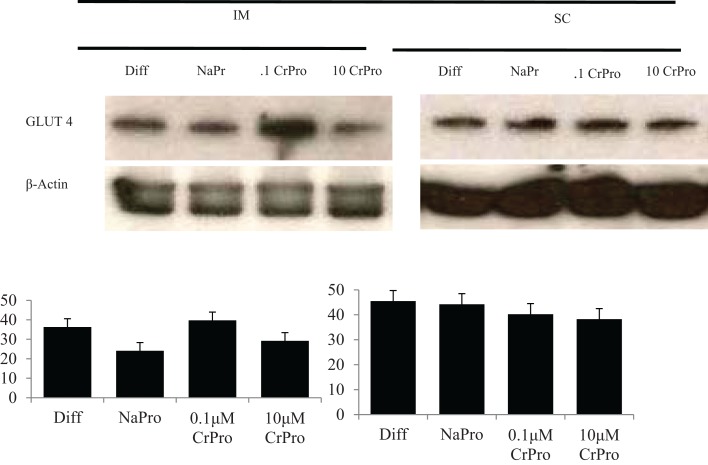
**Effects differentiation media (Diff), chromium propionate (CrPro), and sodium propionate (NaPro) on glucose transporter type 4 (GLUT4) protein abundance (*n* ***=*** 3)**. Protein was isolated from bovine intramuscular and subcutaneous adipocytes after 96 h of treatment, and protein abundance was determined through western blot analysis. Bars represent the standard error of the difference between the treatment means.

No difference was detected between differentiation media and CrPro-treated adipocytes and in AMPKα mRNA abundance for either the IM or SC adipocytes at 96, 120, or 144 h (Figures 4 and 5). At 144 h in SC cultures, adipocytes treated with differentiation media plus 0.1 or 1 μM CrPro had a greater PPARγ mRNA abundance (*P* < 0.01) compared to adipocytes treated with differentiation media (Figure 5). However, the highest dose was not different from negative control. Again, these data indicated that a lower dose of CrPro may stimulate differentiation of SC preadipocytes while IM preadipocytes require a higher dose. The relative abundance of GLUT 4 mRNA at 144 h was not different between differentiation media and CrPro-treated cultures in either IM or SC adipocytes (Figures 4 and 5). Additionally, there was no effect on GPR43, and SCD mRNA relative abundance due to CrPro addition in either IM or SC adipocytes at 144 h (Figures 4 and 5). However, there was increased (*P* < 0.01) PPARγ mRNA relative abundance when differentiation media was added to the cultures, which indicated that cells underwent normal differentiation in our culture system. In SC adipocytes treated with CrPro or NaPro, no significant differences in GLUT4 protein were detected (Figure 6). However, 0.1 μM CrPro treatment tended (*P* = 0.10) to increase the GLUT4 protein level in IM adipocytes compared to NaPro treatment (Figure 6).

**Figure 7:**
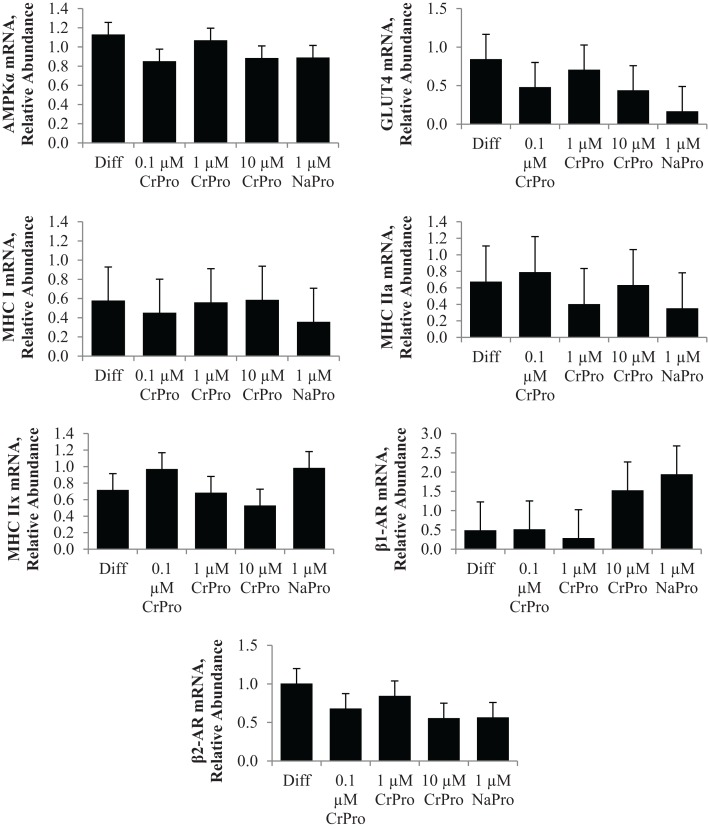
**Effect of differentiation media (Diff), chromium propionate (CrPro), and sodium propionate (NaPro) on adenosine monophosphate-activated protein kinase (AMPK***α***), glucose transporter type 4 (GLUT4), myosin heavy chain (MHC)-I, MHC-IIa, and MHC-IIX, ***β***1-adernergic receptor (AR), and ***β***2-AR mRNA levels in bovine satellite cells (*n* ***=*** 3)**. Bars represent the standard error of the difference between the treatment means. There was no effect (*P* > 0.10) of treatment on the mRNA expression of AMPKα, GLUT4, MHC I, MHC-IIa, MHC-IIx, β1-AR, or β2-AR.

**Figure 8:**
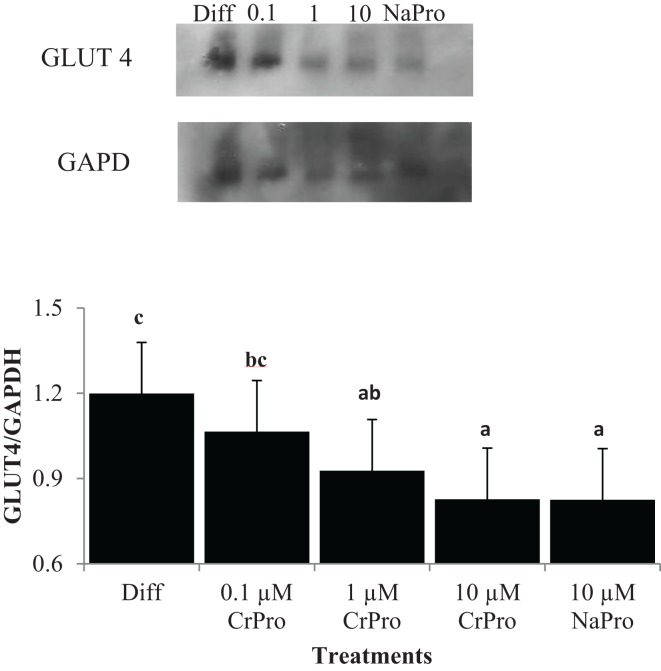
**Effect of differentiation media (Diff), chromium propionate (CrPro), and sodium propionate (NaPro) on glucose transporter type 4 (GLUT4)/glyceraldehyde 3-phosphate dehydrogenase (GAPDH) protein abundance (*n* ***=*** 4)**. Protein was isolated from bovine satellite cells after 72 h of treatment and protein abundance was determined through western blot analysis. Bars shown represent the standard error of the difference between the treatment means. Bars with different letters differ (*P* < 0.01).

In the third experiment, BSCs were used to determine the effect of chromium on muscle cells. No differences (*P* > 0.10) in mRNA relative abundance were observed when CrPro or NaPro treatments were added to the media for AMPKα, GLUT 4, MHC I, MHC-IIa, MHC-IIx, β1-AR, and β2-AR expression (Figure 7). However, Western blot analysis of BSC indicated that the protein abundance of GLUT4 relative to GAPDH significantly decreased (*P* < 0.01) in a dose-dependent manner as increased concentrations of chromium were added to the BSC culture media (Figure 8). These data are very interesting and suggest a potential negative feedback mechanism on GLUT4 protein due to enhanced efficiency of glucose uptake regulated by Cr in a muscle cell culture system.

## Discussion

The results from this study indicated that the treatment of cells with CrPro altered mRNA levels of adipogenic and metabolic markers, as well as inducing morphological changes in bovine muscle cells and preadipocytes. Chromium addition had divergent effects in the three different cell types. In IM adipocytes, the NaPro treatment had less lipid droplets compared to the differentiation treatment indicating that chromium itself enhances adipocyte differentiation rather than propionate. Oil-red-O stained images of these treatments indicated that multilocular lipid droplets accumulated in IM preadipocytes compared to the unilocular lipid droplets that accumulated in the SC preadipocytes at 96 h. The different distribution of lipid droplets indicates a basic morphological difference between IM and SC adipocytes and shows that the cell culture model was accurate in distinguishing the two cell types. Smith and Crouse demonstrated that IM and SC adipose tissues had metabolic differences (6). They also showed that the proportion of carbon provided by glucose for bovine adipocyte development decreased from 65% in IM adipose tissue and from 22% in SC adipose tissue. These findings suggested that glucose is a more important source for IM adipose development than SC adipose development. In contrast, acetate is a more important source for SC adipose tissue development than for IM adipose tissue development. The morphological differences between IM and SC adipocytes may be regulated by these metabolic differences. In IM adipoctyes, the number of lipid droplets increased as the dose of CrPro was increased. Interestingly, in SC adipocytes, the number of lipid droplets also increased as the dose of CrPro was increased up to 1 μM CrPro. However, SC adipocytes treated with 10 μM CrPro had less lipid droplets that SC adipocytes treated with 1 μM CrPro. This suggests that in SC adipocytes, CrPro concentration above 1 μM CrPro may begin to have a negative effect on adipocyte development. The data also suggested that a higher dose is required in IM preadipocyte to get maximal glucose uptake in these cells. The GLUT4 data supported this concept due to an apparent positive effect of CrPro on Glut4 protein in IM preadipocytes but no effect in SC preadipocytes. Taken together, these data indicated that IM adipocytes are more sensitive to Cr from a glucose regulation standpoint as compared to SC adipocytes. Biologically, this makes sense since IM cells require glucose as the energy substrate whereas SC adipocytes can synthesize lipids from acetate (6).

Adipogenic markers, such as PPARγ, were also differentially expressed for IM compared to SC adipocytes. Peroxisome proliferator-activated receptor γ is involved in gene regulation and its transcriptional activity is stimulated by insulin (7). The upregulation of PPARγ that results from this stimulation can increase the cellular uptake of fatty acids and enhance adipocyte development (8). In the first experiment, treatment with 10 μM CrPro upregulated PPARγ mRNA expression in IM adipocytes at 96 h compared to all other treatments. This mirrored the morphological changes seen in the IM adipocytes where an increasing dosage of CrPro resulted in an increase in lipid droplet number. In the second experiment, 0.1 and 1 μM CrPro-treated SC adipocytes had a higher PPARγ mRNA expression than the differentiated control SC adipocytes at 144 h. However, at 144 h in the SC adipocytes, the 10 μM CrPro-treated samples were not different from controls in PPARγ mRNA expression. Again, this mirrored the morphological changes observed in SC adipocytes. In the SC adipocytes, an increasing dose of CrPro, up to 1 μM, is beneficial to lipid droplet number. However, CrPro treatment in excess of 1 μM appears to cause a decline in lipid droplet number and as a result, could hinder adipocyte development. Together, these effects of CrPro on PPARγ and lipid droplet number suggested that higher concentrations of CrPro may be used to enhance IM adipocyte development without augmenting SC adipocyte development.

Adenosine monophosphate-activated protein kinase α, which regulates cellular energy balance, is an important enzyme in regulating lipid metabolism not only in adipocytes but also in BSC. Activation of AMPKα in muscle and adipose tissue triggers metabolic changes, such as the switch from storing to consuming fats and carbohydrates. Phosphorylation of AMPKα has been shown to be positively correlated with GLUT4 activity, which regulates glucose entry into muscle and adipose tissue (9). In experiment 1 at 96 h, relative AMPKα mRNA levels isolated from IM adipocyte increased with the 10 μM CrPro treatment but were not different from differentiated controls in SC adipocytes. This indicated that the level of AMPKα can be regulated by treatment with CrPro. These data suggest that enhancement of AMPKα by CrPro treatment may improve glucose uptake in IM adipocytes.

In the third experiment, a comparison of the BSC micrographs of 0.1 μM CrPro to either 1 or 10 μM CrPro revealed that there was a qualitative effect of CrPro on BSC cultures. The BSC cultures treated with CrPro appeared to have a greater myotube number and diameter compared to the control cultures. The difference in myotube size may be a result of increased energy or glucose uptake by those cultures, resulting in larger myotubes. *In vivo*, these differences would be manifested as changes in postnatal muscle hypertrophy. Again, this would be positive to muscle growth, especially during the feeding of a beta-adrenergic agonist, the last part of the feeding period. This combination of larger myotubes and the decreased GLUT4/GAPDH protein ratio may suggest that CrPro is causing muscle tissue to be much more efficient at glucose uptake, so the cell actually needs less GLUT4 to take up the same amount of glucose. Since increased myotube number and diameter were still detected, the glucose transporters may be also more efficient with treatment of CrPro. This would explain why mRNA did not change and protein abundance decreased, but morphological effects were still observed. However, the IM preadipocytes normally need to compete with skeletal muscle for glucose in ruminants. Our data indicated that CrPro actually stimulated GLUT4 in those cells in means to more effectively compete with skeletal muscle tissue for glucose. In fact, our dose titration work suggested that higher the dose, the more effect on glucose metabolism and lipid filling in IM preadipocytes. In contrast, CrPro had little effect on glucose transport in SC preadipocytes. This makes sense since they do not require glucose to synthesize lipids and can rely on acetate.

Chromium, a trace mineral required for insulin action, regulated mode of action of insulin signaling and has been shown to induce glucose absorbance in the muscle and adipocytes (10). Chromium Pro and Cr methionine have also been reported to inducing glucose uptake and feed intake (11). This study indicated that a dose-dependent Cr supplementation increased glucose level, decreased insulin, and non-esterified fatty acids in the serum. Recent data indicated that pharmacological doses of Cr appear to increased insulin sensitivity in rodent study and the mode of action of Cr could serve in a regulation of second messenger in animals (12). These data also indicated that transferrin is a major agent as physiological Cr transport in bloodstream. The receptor-specific regulation of transferrin is sensitive to insulin and it results in a stimulation of inorganic second messenger in plasma membrane. Therefore, CrPro likely induces glucose utilization through insulin signaling for growing cattle. Our data indicated that a time-dependent treatment of CrPro increased GLUT4 levels in IM adipocyte cultures. These GLUT4 data indicated that treatment of CrPro may be regulating the early phase of IM adipocyte differentiation. The level of GLUT4 was steadily increased in both IM and SC adipocytes during the late phase of adipocyte differentiation. However, bovine satellite cells treated with CrPro in this study showed no effect on GLUT4 mRNA expression and had decreased GLUT4/GAPDH protein ratio in a dose-dependent fashion. The variation in these results between adipocytes and muscle satellite cells indicated that CrPro has differential effects on different tissues. In essence, the CrPro decreased GLUT4 protein levels in muscle cell cultures (Figure 8) indicating that those cells have increased efficiency of glucose uptake due to exposure to increased levels of CrPro. In contrast, each of the two adipogenic lines had opposing responses to the CrPro. It appeared that CrPro had the most stimulative effect of GLUT4 response in the IM adipocytes as compared to SC adipocytes. This suggests opportunities to potentially augment marbling in beef cattle fed elevated levels of CrPro during the finishing period. In addition, skeletal muscle growth could be further enhanced in cattle being fed both a beta-adrenergic agonist and CrPro the last 20–40 days on feed.

## Conflict of Interest Statement

The authors declare that the research was conducted in the absence of any commercial or financial relationships that could be construed as a potential conflict of interest.

## Funding

Supported in part by funding from Kemin Animal Nutrition and Health North America, Des Moines, IA 50317 and partially supported by “Cooperative Research Program for Agriculture Science & Technology Development (PJ00941401)” RDA, Republic of Korea. The Gordon W. Davis Regent’s Chair in Meat and Muscle Biology Endowment at Texas Tech University provided funding to support this research.
